# Supplementary material for: Splice-Junction-Based Mapping of Alternative Isoforms in the Human Proteome
Source: Cell Rep. Author manuscript; Available in PMC 2020 Jan 15. (PMC6961840; doi:10.1016/j.celrep.2019.11.026)
Supplement: 3 [file NIHMS1546469-supplement-3.zip › DF2/PXD006675/SmoothMuscleCells-75-Q96D71-SPSMPSLNQTWPELNQSSEDTAIVHPVPIR.pdf]

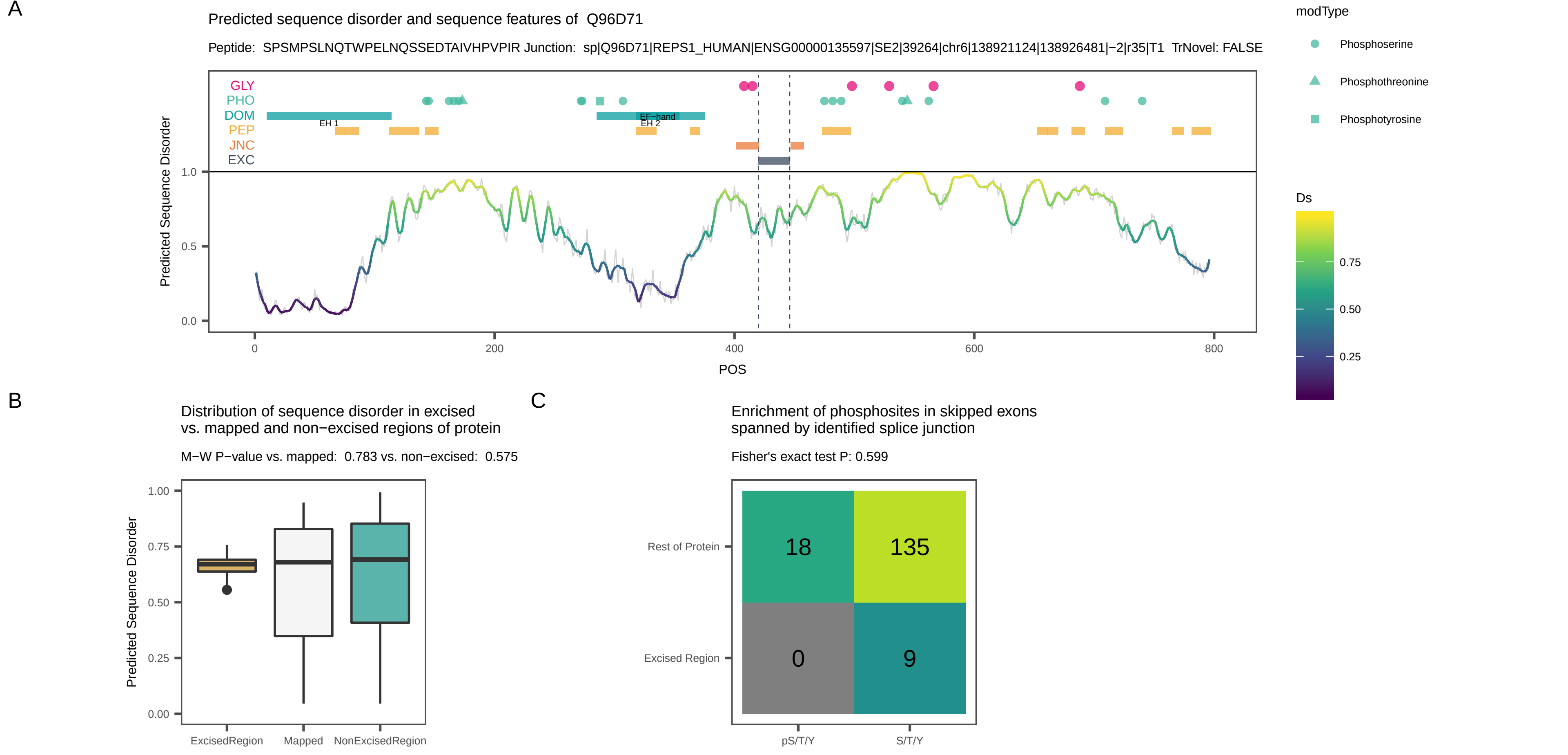
A
Predicted sequence disorder and sequence features of Q96D71
Peptide: SPSMPSLNQTWPELNQSSEDTAIVHPVPIR Junction: sp|Q96D71|REPS1_HUMAN|ENSG00000135597|SE2|39264|chr6|138921124|138926481|−2|r35|T1 TrNovel: FALSE
GLY
PHO
DOM
PEP
JNC
EXC
EH 1
EF-hand
EH 2
Predicted Sequence Disorder
1.0
0.5
0.0
0
200
400
600
800
POS
modType
Phosphoserine
Phosphothreonine
Phosphotyrosine
Ds
0.75
0.50
0.25
B
Distribution of sequence disorder in excised
vs. mapped and non−excised regions of protein
M−W P−value vs. mapped: 0.783 vs. non−excised: 0.575
Predicted Sequence Disorder
1.00
0.75
0.50
0.25
0.00
ExcisedRegion
Mapped
NonExcisedRegion
C
Enrichment of phosphosites in skipped exons
spanned by identified splice junction
Fisher's exact test P: 0.599
Rest of Protein
Excised Region
18
135
0
9
pS/T/Y
S/T/Y
